# Robot-Assisted Total Gastrectomy for Gastric Adenocarcinoma and Proximal Polyposis of the Stomach: A Case Report

**DOI:** 10.7759/cureus.109578

**Published:** 2026-05-25

**Authors:** Takafumi Ihashi, Hironori Iwasaki, Akifumi Kawahito, Tsuguharu Asato, Hiroshi Yokomizo

**Affiliations:** 1 Department of Surgery, Japanese Red Cross Kumamoto Hospital, Kumamoto, JPN; 2 Department of Pathology, Japanese Red Cross Kumamoto Hospital, Kumamoto, JPN

**Keywords:** apc promoter 1b, fundic gland polyposis, gapps, gastric adenocarcinoma, hereditary gastric cancer, robotic gastrectomy, spleen preservation, splenic hilar dissection, total gastrectomy

## Abstract

Gastric adenocarcinoma and proximal polyposis of the stomach (GAPPS) is a rare hereditary syndrome characterized by fundic gland polyposis and an increased risk of gastric adenocarcinoma. We report a case of gastric cancer associated with clinically suspected GAPPS that was successfully treated with robot-assisted total gastrectomy. A 26-year-old woman presented with epigastric pain, and upper gastrointestinal endoscopy revealed diffuse fundic gland polyposis extending from the gastric fundus to the lower gastric body. Biopsy specimens demonstrated well-differentiated tubular adenocarcinoma. No polyps were identified in the gastric antrum, duodenum, or colon. Based on the characteristic endoscopic findings, family history, and exclusion of other hereditary polyposis syndromes, the patient was clinically suspected to have GAPPS. Contrast-enhanced computed tomography suggested possible splenic hilar lymph node metastasis, and robot-assisted total gastrectomy with D2 lymphadenectomy and spleen-preserving splenic hilar lymph node dissection was performed. The postoperative course was uneventful, and the patient was discharged on postoperative day seven. Histopathological examination demonstrated multiple intramucosal well-differentiated tubular adenocarcinoma lesions without lymph node metastasis. The patient remains free of recurrence two years and six months after surgery. GAPPS carries a substantial risk of malignant transformation at a relatively young age, and accurate endoscopic evaluation may be challenging. In young patients requiring total gastrectomy, robot-assisted surgery may represent a feasible minimally invasive option that facilitates precise dissection in technically demanding procedures.

## Introduction

Gastric adenocarcinoma and proximal polyposis of the stomach (GAPPS) is an autosomal dominant hereditary disorder characterized by fundic gland polyposis predominantly affecting the gastric fundus and body. It was first described in 2012 [1], and pathogenic variants in the promoter 1B region of the APC gene have been identified as the underlying cause [2]. GAPPS is extremely rare, with only several dozen families reported worldwide [3], and its penetrance and natural history remain incompletely understood. We report a case of gastric cancer associated with a clinically suspected GAPPS phenotype that was successfully treated with robot-assisted total gastrectomy.

## Case presentation

A 26-year-old woman presented with epigastric pain. Upper gastrointestinal endoscopy performed at a previous hospital revealed multiple gastric polyps, and she was referred to our institution for further evaluation. Detailed examination demonstrated fundic gland polyposis, and biopsy specimens confirmed adenocarcinoma. She was subsequently referred to our department for surgical treatment. She had no history of proton pump inhibitor (PPI) use. Her family history was notable for colorectal cancer in her paternal grandfather, gastric cancer in her paternal great-grandfather, and gastric polyposis in her mother. 

**Figure 1:**
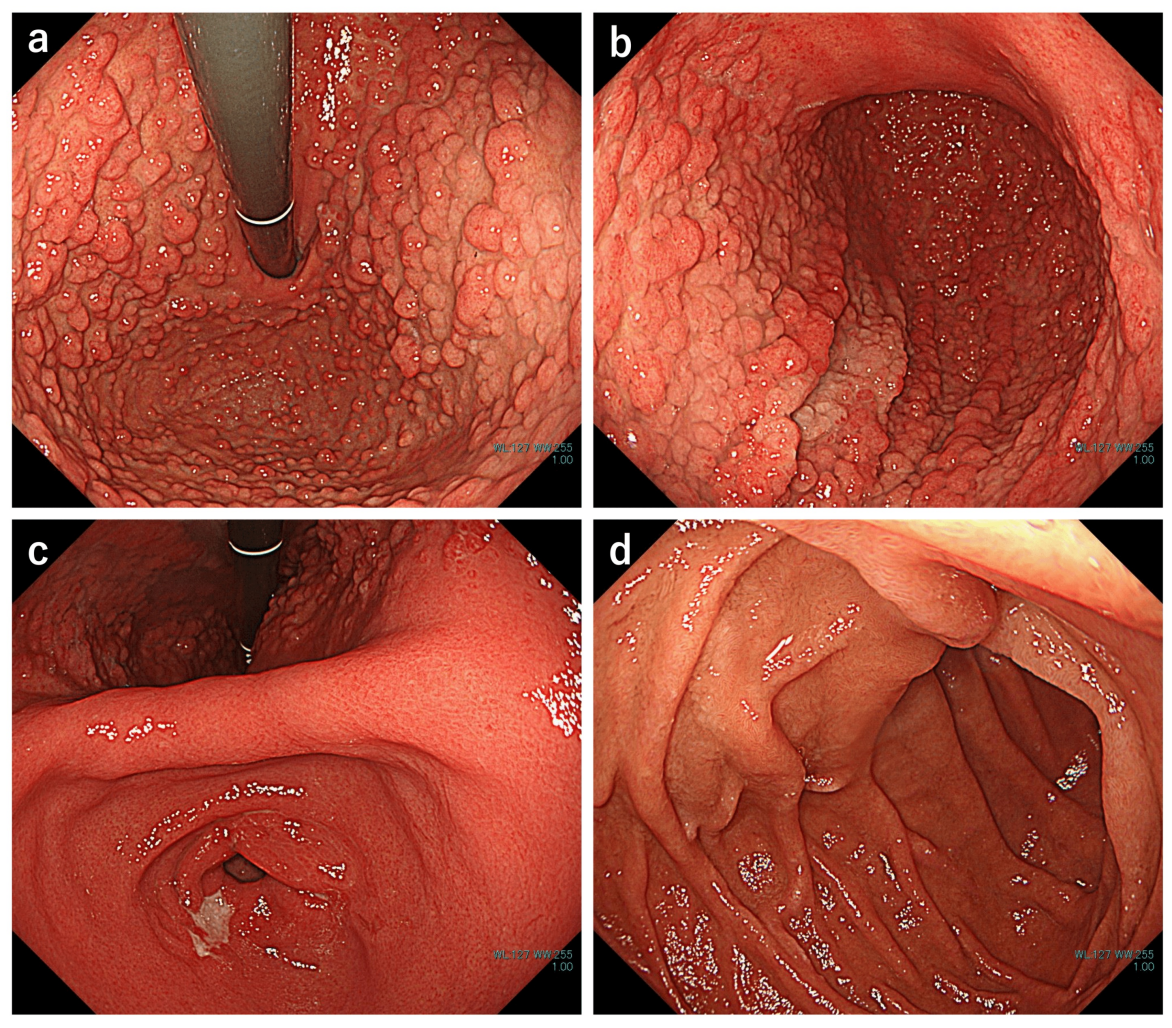
Upper gastrointestinal endoscopic findings (a, b) Diffuse polyposis extending from the gastric fundus to the lower gastric body. (c, d) No polyps were observed in the gastric antrum or duodenum.

**Figure 2:**
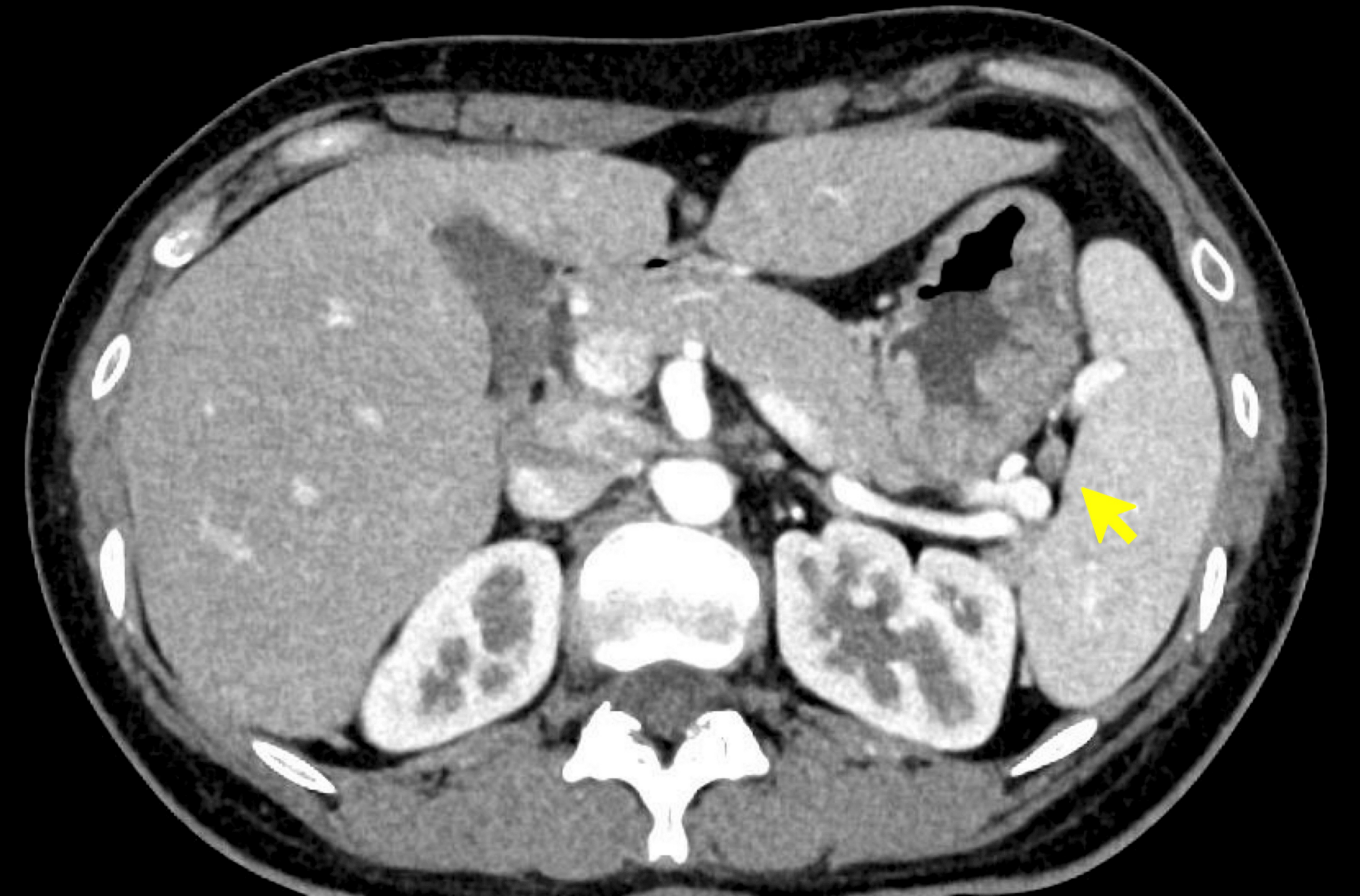
Axial contrast-enhanced CT scan Enlarged splenic hilar (#10) lymph node (yellow arrow).

On admission, her weight was 48.6 kg, body mass index was 19.2 kg/m², and Eastern Cooperative Oncology Group performance status was 0. Laboratory findings, including tumor markers, were within normal limits. Upper gastrointestinal endoscopy revealed innumerable fundic gland polyps extending from the gastric fundus to the lower gastric body (Figures 1a, 1b). Biopsies obtained from multiple polyps on the greater curvature of the lower gastric body demonstrated well-differentiated tubular adenocarcinoma. Although a well-demarcated ulcerative lesion was observed near the pylorus, biopsy specimens showed no evidence of malignancy. No polyps were observed in the gastric antrum or duodenum (Figures 1c, 1d). Colonoscopy revealed no abnormal findings, including colorectal polyps. Contrast-enhanced computed tomography (CT) demonstrated no obvious primary gastric lesion. However, enlargement of multiple regional lymph nodes, including splenic hilar lymph nodes, was observed (Figure 2). No ascites, pleural effusion, or distant metastases were identified.

**Figure 3:**
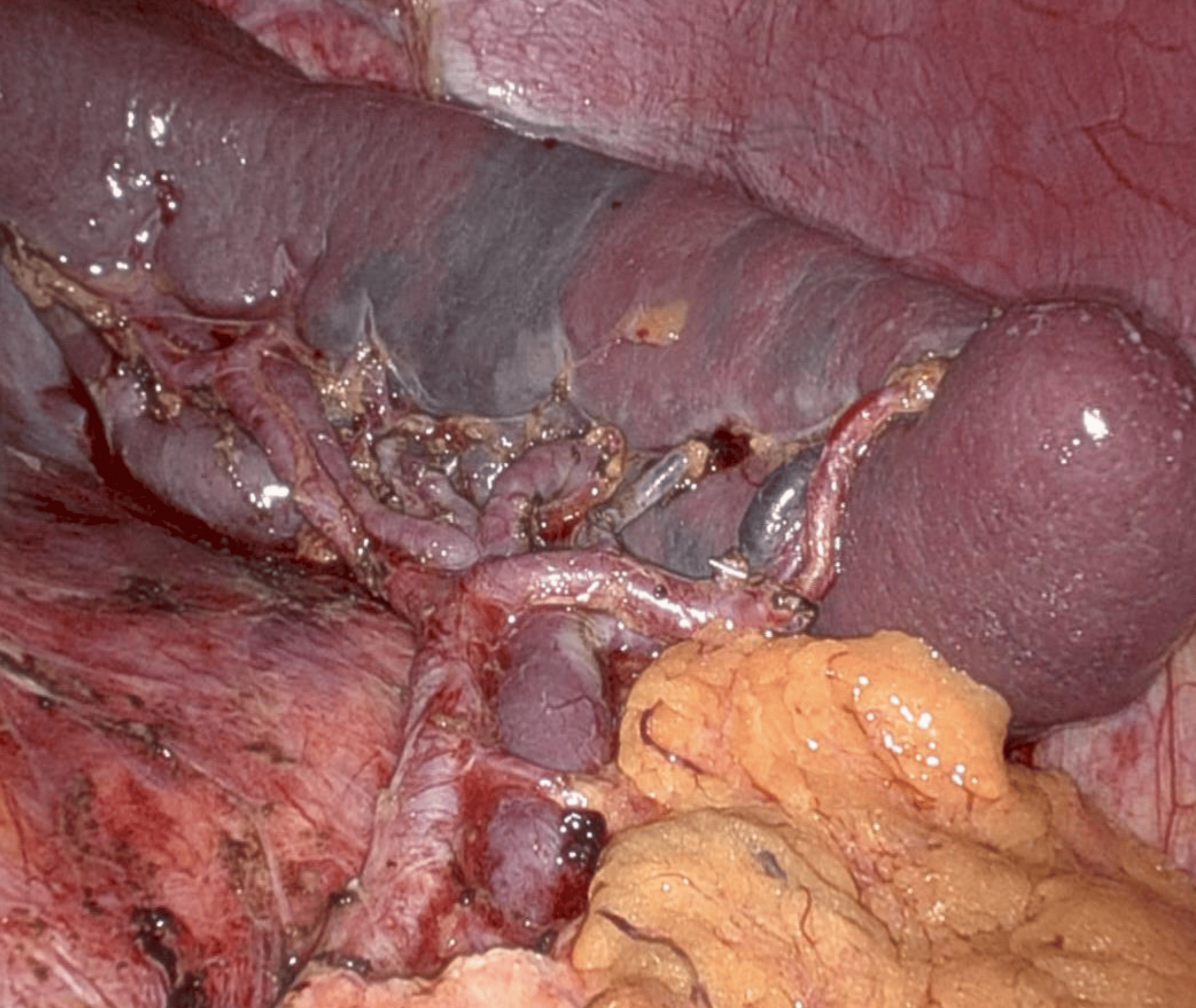
Intraoperative findings Robot-assisted spleen-preserving splenic hilar lymph node dissection. Precise dissection was performed around the complex splenic hilar vasculature and pancreatic tail while preserving the spleen and hilar vessels.

She was diagnosed with cT1aN2M0 Stage IIA according to the Japanese Classification of Gastric Carcinoma [4]. Although the primary lesions appeared limited to the mucosa, lymph node metastasis could not be excluded preoperatively based on imaging findings, including splenic hilar lymph node enlargement. Robot-assisted total gastrectomy with D2 lymphadenectomy, splenic hilar lymph node dissection, and Roux-en-Y reconstruction was performed. A standard five-port robotic approach was used with the patient in the reverse Trendelenburg position. Splenic hilar dissection was carefully performed around the splenic vessels and pancreatic tail while preserving the spleen. Because splenic hilar lymph node metastasis could not be excluded preoperatively and the patient was young, spleen-preserving dissection was selected to minimize surgical invasiveness and avoid the risk of overwhelming post-splenectomy infection (Figure 3). Operative time was 7 hours and 38 minutes, and intraoperative blood loss was 5 mL.

Her postoperative course was uneventful. Oral solid food intake was initiated on postoperative day three, and she was discharged home without complications on postoperative day seven. At two years and six months after surgery, she remains free of recurrence or metastasis and has maintained a favorable clinical course.

**Figure 4:**
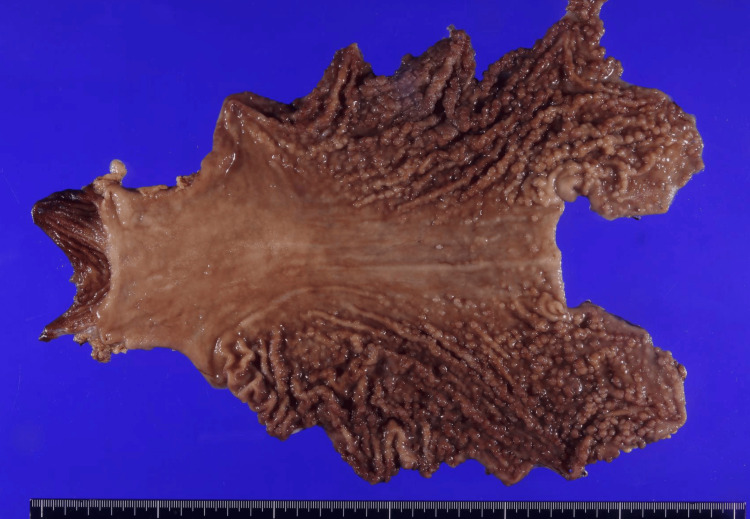
Gross appearance of the resected specimen Gastric polyposis predominantly involving the fundic gland region.

**Figure 5:**
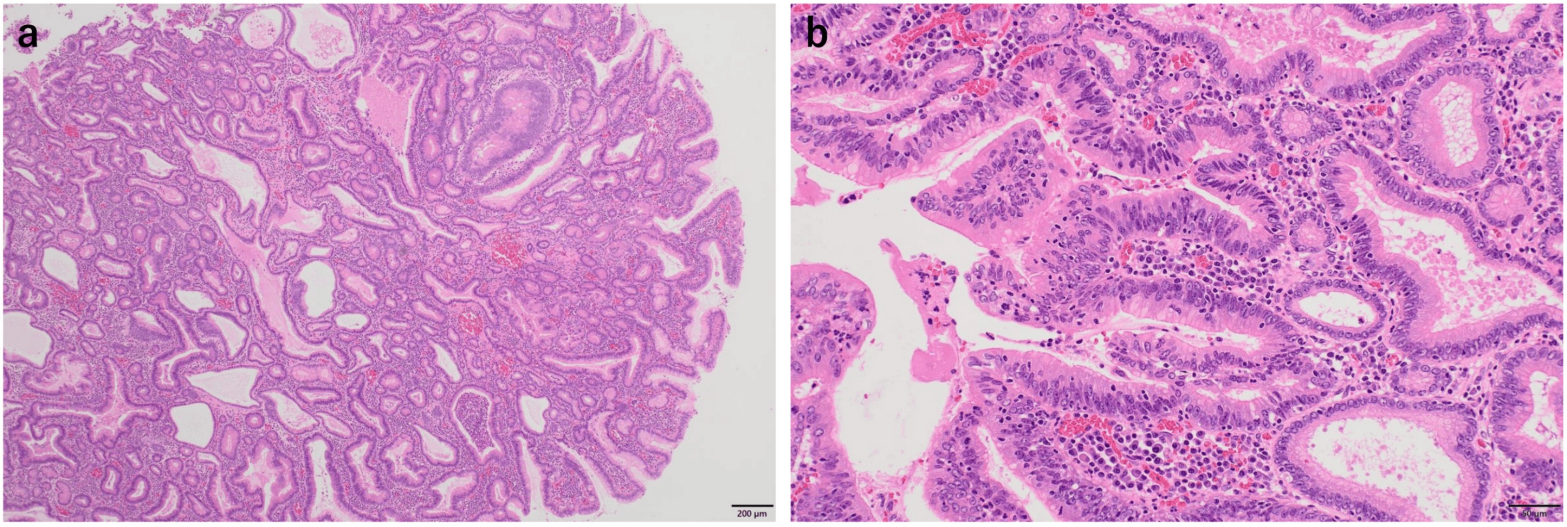
Histopathological findings (a) Hyperplasia of the foveolar epithelium and irregular cystic dilatation of glandular structures corresponding to fundic gland polyps (hematoxylin and eosin staining, ×40). (b) Well-differentiated tubular adenocarcinoma (tub1) showing nuclear pleomorphism, stratification, and loss of polarity arising in a background of atypical fundic gland-type epithelium (hematoxylin and eosin staining, ×200).

Histopathological examination revealed gastric polyposis predominantly involving the fundic gland region (Figure 4). Diffuse hyperplastic and dysplastic changes were observed in the fundic glands and foveolar epithelium (Figure 5a). Multiple intramucosal lesions corresponding to well-differentiated tubular adenocarcinoma were identified, with indistinct borders between dysplastic and carcinomatous lesions (Figure 5b). A total of 50 lymph nodes were retrieved, and no lymph node metastases were detected. The final pathological diagnosis according to the Japanese Classification of Gastric Carcinoma was pT1aN0M0, Stage IA.

Informed consent for publication was obtained from the patient. According to the institutional policy of our hospital, ethics committee approval was waived for single case reports. 

## Discussion

GAPPS is a rare autosomal dominant hereditary syndrome characterized by gastric adenocarcinoma and dysplastic lesions arising in the background of fundic gland polyposis. It was first proposed by Worthley et al. in 2012. Proposed diagnostic criteria include: (1) fundic gland polyps localized to the gastric fundus and body without polyposis in the duodenum or colon; (2) more than 100 proximal gastric polyps in the proband or more than 30 proximal gastric polyps in a first-degree relative; (3) predominance of fundic gland polyps with areas of dysplasia, or a family member with dysplastic fundic gland polyps or gastric adenocarcinoma; (4) an autosomal dominant inheritance pattern; and (5) exclusion of other hereditary polyposis syndromes and use of PPIs [1]. Subsequently, point mutations in the promoter 1B region of the APC gene were identified as the genetic cause of GAPPS [2]. In the present case, the patient demonstrated diffuse fundic gland polyposis localized to the proximal stomach with biopsy-proven adenocarcinoma, while no polyps were identified in the antrum, duodenum, or colon. In addition, her mother had gastric polyposis, and there was no history of PPI use or clinical findings suggestive of other hereditary polyposis syndromes, including familial adenomatous polyposis, juvenile polyposis syndrome, Peutz-Jeghers syndrome, or Cowden syndrome. Therefore, the patient was clinically suspected to have GAPPS.

A limitation of this case is the absence of genetic confirmation. Because gastric cancer had already been diagnosed during the initial evaluation and total gastrectomy was indicated regardless of the genetic findings, preoperative genetic testing was not performed. Postoperatively, genetic testing was repeatedly recommended to the patient and her family members; however, it was not pursued. Nevertheless, the characteristic endoscopic distribution, family history, absence of extra-gastric polyposis, and lack of PPI exposure strongly supported the clinical diagnosis of GAPPS. This case also highlights the practical challenges associated with genetic counseling and testing in hereditary tumor syndromes.

GAPPS is associated with a substantial risk of malignant transformation, and gastric cancer may develop at a relatively young age compared with sporadic gastric cancer [5]. In addition, several reports have demonstrated the limitations of endoscopic surveillance in GAPPS. Repak et al. described a patient who developed fatal advanced gastric cancer with liver metastases and peritoneal dissemination despite undergoing surveillance endoscopy without apparent dysplasia or malignancy [6]. Okamoto et al. also reported that the median interval from cancer diagnosis to death in patients with GAPPS was only seven months (range: approximately 20 days to five years) once distant metastasis had developed [5]. Therefore, prophylactic gastrectomy has increasingly been discussed for patients with GAPPS, particularly in those with dysplastic lesions [7-9]. The 2024 European Hereditary Tumour Group guideline recommends prophylactic total gastrectomy for GAPPS patients with high-grade dysplasia [10]. On the other hand, total gastrectomy is associated with risks of severe complications such as anastomotic leakage and pancreatic fistula, and long-term postoperative issues, including reduced oral intake, dumping syndrome, and reflux symptoms, may significantly impair physical function and quality of life. A 2025 review by Fugăretu et al. reported that prophylactic gastrectomy had been performed in approximately half of previously reported GAPPS cases, with a mean age at surgery of 42 years [11]. Since patients with GAPPS are often relatively young and prophylactic gastrectomy may be considered even before cancer progression, minimizing surgical invasiveness and preserving postoperative function are particularly important. Therefore, decision-making regarding prophylactic gastrectomy in GAPPS should balance the high risk of malignant transformation against postoperative quality of life, especially in young patients.

Although preoperative imaging suggested splenic hilar nodal metastasis, final pathological examination revealed no lymph node metastases. In retrospect, the discrepancy between radiologic N2 disease and pathologic N0 status suggests that the preoperative lymph node enlargement may have represented reactive changes rather than true metastases. However, because accurate preoperative nodal staging in GAPPS-associated gastric cancer remains difficult, and splenic hilar nodal involvement could not be excluded radiologically, D2 lymphadenectomy with splenic hilar dissection was considered an oncologically acceptable approach.

Robot-assisted gastrectomy has recently become more widely adopted because of advantages such as stable three-dimensional visualization, precise instrument control, and greater dexterity provided by articulated instruments. Compared with laparoscopic surgery, robotic surgery has been associated with reduced intraoperative blood loss and lower rates of postoperative complications such as pancreatic fistula [12,13]. In the present case, robot-assisted total gastrectomy with spleen-preserving splenic hilar lymph node dissection was performed. The relatively long operative time may have reflected the technical complexity of spleen-preserving splenic hilar dissection and the learning curve associated with robotic gastric surgery. Because splenic hilar dissection requires meticulous manipulation around the complex vascular anatomy, robotic assistance was particularly useful for achieving precise dissection while preserving the spleen. In this young patient, minimizing surgical invasiveness while maintaining oncological radicality was considered especially important.

Although reports of robotic gastrectomy for GAPPS remain limited, a PubMed search using the keywords “GAPPS,” “robotic,” and “gastrectomy” identified only 13 reported cases between 2012 and November 2025, including five from Japan [14-16]. Because of the rarity of this disease, further accumulation of cases is needed to clarify the optimal surgical strategy and long-term outcomes in patients with GAPPS.

## Conclusions

We reported a case of GAPPS-associated gastric cancer successfully treated with robot-assisted total gastrectomy and spleen-preserving splenic hilar lymph node dissection. Because GAPPS carries a substantial risk of malignant transformation at a relatively young age and may be difficult to evaluate accurately by endoscopic surveillance alone, early recognition and appropriate surgical intervention are important. In young patients requiring total gastrectomy, robot-assisted surgery may represent a feasible minimally invasive approach that facilitates precise dissection in technically demanding procedures.
